# Imaging of Mitral Valve Prolapse: What Can We Learn from Imaging about the Mechanism of the Disease?

**DOI:** 10.3390/jcdd2030165

**Published:** 2015-07-10

**Authors:** Ronen Durst, Dan Gilon

**Affiliations:** Heart Institute, Ein Kerem Campus, Hadassah Hebrew University Medical Center, POB 12000, 92110 Jerusalem, Israel; E-Mail: dangi@ekmd.huji.ac.il

**Keywords:** mitral valve prolapse, imaging, myxomatous degeneration, fibroelastic deficiency

## Abstract

Mitral valve prolapse (MVP) is the most common mitral valve disorder affecting 2%–3% of the general population. Two histological forms for the disease exist: Myxomatous degeneration and fibroelastic disease. Pathological evidence suggests the disease is not confined solely to the valve tissue, and accumulation of proteoglycans and fibrotic tissue can be seen in the adjacent myocardium of MVP patients. MVP is diagnosed by demonstrating valve tissue passing the annular line into the left atrium during systole. In this review we will discuss the advantages and limitations of various imaging modalities in their MVP diagnosis ability as well as the potential for demonstrating extra associated valvular pathologies.

## 1. History of MVP Diagnosis

**Figure 1:**
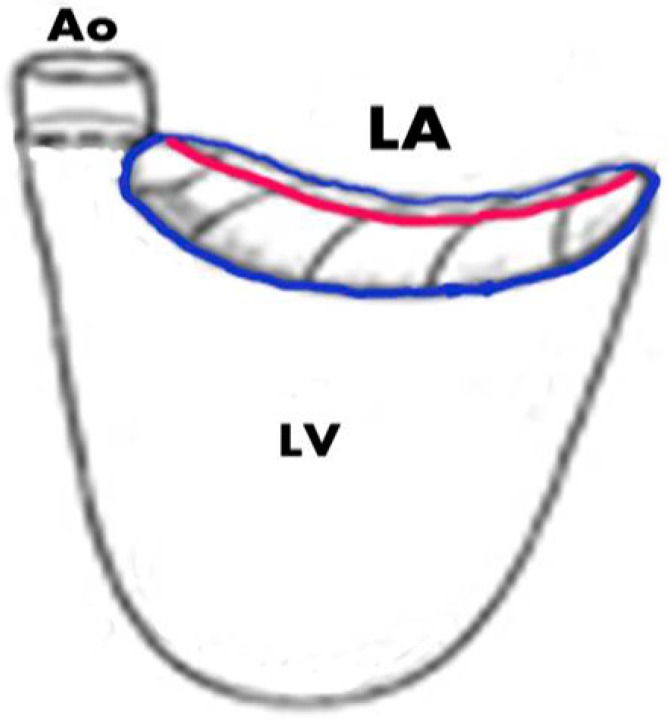
Scheme of the mitral valve anatomy with the saddle shaped annulus (**blue**) and the plane of the valve as seen in para-sternal long axis view on echo (**pale red**) showing the two high points of the annulus. Ao = aorta, LV = left ventricle, LA = left atrium.

**Figure 2:**
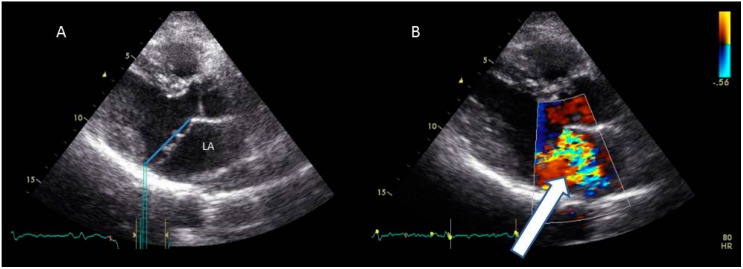
A parasternal long axis view of a prolapsing valve. Panel **A** shows the prolapsing valve relative to the annular line (**blue line**). Panel **B** shows color Doppler through the prolapsing valve demonstrating a severe jet of regurgitation (**arrow**). LA = left atrium.

MVP is a common cardiovascular disorder, originally described in the 1960s and is considered today the most common cause of mitral regurgitation [1,2,3]. Barlow and Bosman described a constellation of clinical findings consisting of non-ejection systolic clicks and a late systolic murmur, T-wave abnormalities, and systolic aneurysmal billowing of the posterior mitral leaflet into the left atrium on left ventriculography [4]. MVP is defined by the abnormal relationship of the mitral leaflets to their surrounding structures as diagnosed by imaging. In early studies, MVP prevalence was considered to be between 5% and 15%, with some estimates even as high as 35% [5,6,7,8,9,10]. These studies relied on auscultation and single-dimensional M-mode echo techniques, which provide only a limited appreciation of cardiac anatomy and produce varying results depending on the orientation of the ultrasound transducer on the chest [11,12,13,14]. Despite the improved spatial appreciation and view standardization achieved with two-dimensional echocardiography, similarly high diagnostic rates could still be obtained based on the assumption that the mitral annulus is planar, and that leaflet displacement relative to the annulus in any view is abnormal [15,16,17]. Three-dimensional echocardiographic pioneering studies by Levine and colleagues have shown that the mitral annulus is not a flat plane but rather has a saddle-like shape with high points positioned anteriorly and posteriorly and low points medially and laterally (Figure 1) [16,17,18,19]. Recognition of the saddle shape has significantly increased the specificity of diagnostic criteria without loss of sensitivity. Commonly today, a displacement of 2 mm or above of the leaflet above the annular plane in parasternal long axis view is considered diagnostic (Figure 2A) [4,20]. The three-dimensional understanding of the mitral valve shape and the new more accurate MVP diagnostic criteria have reduced the prevalence of the disease in various series to 2%–3% of the general population [3,14]. Strengthening the accuracy of this criterion is the fact that it has been used in genetic studies to successfully clone MVP segregating allelic loci [21,22]. The definition of MVP is based on demonstrating the anatomical relation between mitral valve leaflet excursion into the left atrium relative to the annulus. Often, pathologies are found in other valve as well. Sometimes the tricuspid valve demonstrates prolapsing segments and leakage, although criteria for defining tricuspid valve prolapse are currently lacking. In some instances, aortic valve pathologies in families segregating MVP were reported [23,24,25]. This relation can be demonstrated by non-invasive imaging. Imaging can also provide an insight into the pathology associated with valve structure as well as potential myocardial abnormalities. The purpose of this paper is to review the advantages and limitations of the various imaging modalities in diagnosing MVP and related pathologies.

## 2. Echocardiography

Degenerative processes resulting in regurgitation of the mitral valve have two main phenotypes: Diffuse myxomatous degeneration (or “Barlow disease”), which may present as a genetic disorder, and a markedly different fibroelastic deficiency (FED) [1,26], which might be caused by an accelerated aging process. Barlow disease is characterized by excess connective tissue, with redundant, thickened and yellowish leaflets, marked annulus dilatation, elongated and thin (sometimes ruptured) or thick (frequently calcified) chordae, disrupted collagen and elastic layers, and with excess of acid mucopolyssacharides. Patients with MVP usually present young or at middle aged after a long history of murmur and/or mitral regurgitation (Figure 2B). Familial clustering of MVP has recently been described in the Framingham heart study [27,28]. Myxomatous degeneration is sometimes associated with connective tissure disorders, such as Marfan’s disease, Loeys–Dietz syndrome, Ehlers–Danlos syndrome and Stickler syndrome. It may also be non-syndromic [4]. FED is characterized by decreased connective tissue, deficient in collagen, elastin and proteoglycans; thin, smooth and translucent leaflets without excess tissue and only moderate annulus dilatation; and thin, slightly elongated chordae. Patients frequently present at an older age with chordal rupture and flail after a rather shorter clinical history. While most of the leaflet is thin, localized myxomatous degeneration and thickening occur within the flail scallop, mainly of the posterior leaflet [28,29,30].

Pathological processes in MVP are not limited to the valves only. Pathological studies of MVP patients with sudden death or death from other causes have shown proteoglycan depositions similar to the deposition within the leaflet also in the myocardial tissue, fibrous endocardial plaque depositions, and non-specific interstitial fibrosis [31,32,33,34,35]. These depositions are thought to play a role in the increased tendency of MVP patients for cardiac arrhythmia, sudden death and chest pain [35,36]. Few studies tried to define echocardiographic criteria to differentiate FED from myxomatous degeneration. Because FED patients tend to be of older age, FED is more often associated with other marks of degenerative valve disease such as annular calcification, aortic sclerosis and pupillary muscle calcification [37,38]. In contrast, myxomatous valves usually show billowing of the body of one or both leaflets with prolapse of the margin of either or both leaflets adjacent to the annular ring. The valve is typically large, with elongated and redundant billowing leaflets; leaflets are bulky and thickened. Leaflet thickness often exceeds 3 mm. Another common feature is displacement of the insertion of the posterior leaflet away from the ventricular crest and toward the atrium, creating an out-pouching at the leaflet base [38]. While these features may help clinicians differentiate FED from myxomatous degeneration, studies are needed to define the sensitivity and specificity of echocardiography in discriminating between the two. This is particularly important because FED tends to have a favorable surgical outcome, whereas myxomatous degeneration is probably more likely to have a genetic etiology. First degree relatives of patients with myxomatous mitral valve disease should be screened by echocardiography.

Echocardiography can be used to assess pathologies in other valves. Echocardiographic definition for tricuspid valve prolapse is still lacking. With the advent of three-dimensional imaging, authors have suggested that 3D images are more suitable for defining the prolapsing segment of the tricuspid valve as well as better define the regurgitant orifice area [39].

Echocardiography is the most widely used imaging tool for evaluating cardiac function. For clinicians, left ventricular chamber size as well as function, and regurgitation severity are important for clinical decision making.

## 3. Magnetic Resonance Imaging (MRI)

**Figure 3:**
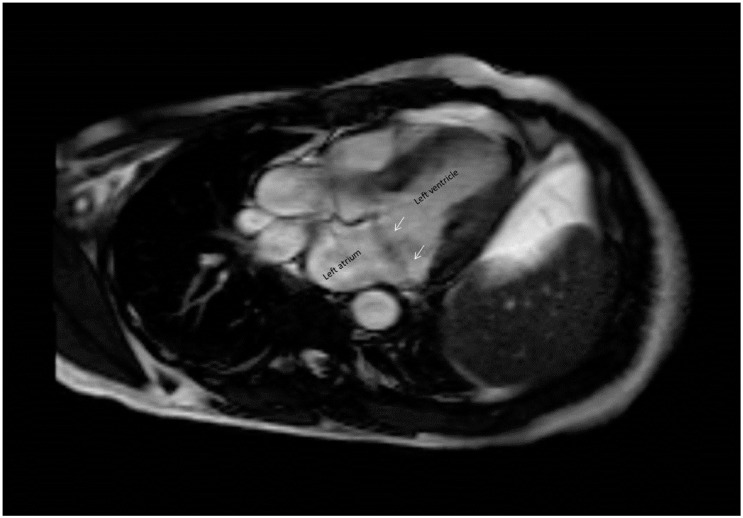
A steady state free precession image on parasternal long axis view of the mitral valve. Note the atrial displacement of both leaflets (arrows).

In recent years cardiac MRI has emerged as a practical, non-invasive tool for cardiac imaging. MRI has several advantages over echocardiography. These include the ability for tissue characterization, unlimited imaging planes, it does not depend on an acoustic window, and it is less invasive then transesophageal echocardiography [40]. Han *et al.* were the first to provide a practical road map for MVP evaluation by MRI [41]. Understanding the saddle shape of mitral valve, Han *et al.* chose a stack of cine SSFP images in the LVOT view spanning the entire mitral valve to evaluate MVP (Figure 3). These imaging planes do not only parallel parasternal long axis and apical long axis of echo in showing the two mitral annulus high points in the a single image, but also enables definition of the prolapsing segments (scallop) [42]. With this approach, the sensitivity and specificity of MVP detection was as high as 100% compared to echocardiography. MRI was also proven to be accurate in evaluating the mitral valve annular dimensions, which may be important whenever a mitral valve prosthesis implantation or repair is planned [43]. While these results are very promising, several topics need to be considered: First, the views used in this study are not routine in every cardiac MRI study [44]. Therefore, the sensitivity specificity reported might only be applied to studies planned a priori for MVP evaluation in which all the relevant image planes have been obtained. Second, one of the key characteristics of myxomatous changes occurring in many patients with MVP is valve thickening. MRI, due to insufficient spatial resolution and volume averaging effect, failed to demonstrate valve thickening.

MRI, like echocardiography, can provide information on the severity of regurgitation. MRI can provide an estimate of the volume of regurgitation and its relation with the overall left ventricular ejection fraction [43]. On the technical level, the volume of mitral regurgitation is quantified indirectly by subtracting the forward flow over the aorta, measured directly with phase-contrast sequence from the left ventricular ejected volume as measured volumetrically. The mitral regurgitant volume is the calculated difference between the LV stroke volume, and the aortic forward stroke volume [45]. 

One of the major advantages of MRI is the ability for tissue characterization. Several studies were able to demonstrate pathological delayed enhancement in the papillary muscle and other regions of the myocardium [41,46,47]. One study demonstrated increased extracellular space by T1 mapping [47]. These delayed enhancement patterns and increased extracellular space may represent fibrosis or proteoglycan depositions or fibrosis. Indeed, myocardial histological analysis of patients demonstrated diffuse fibrosis [47]. The clinical significance of delayed enhancement in MVP patients is unclear. However, in the future, these myocardial changes may prove to correlate with MVP complications such as sudden death or heart failure and therefore may be useful for clinical decision processes and for prognostication.

MRI can provide high-resolution volumetric images of the left ventricle as well as provide quantitative assessment of regurgitant volume. These are important for clinical decision-making and are an added value of MR imaging.

## 4. Computed Tomography (CT)

Computed tomography (CT) of the heart has emerged in recent years as valuable tool for noninvasive imaging of the heart. It provides high-resolution volumetric datasets that can be post-processed to provide views suitable for MVP diagnosis. After acquisition, images in planes demonstrating both superior aspect of the saddle shaped annulus of the mitral valve are generated. The relation between valve leaflets and annulus can be studied, and the degree of leaflet displacement can be measured [48]. An important advantage of CT over echocardiography is that it allows for the visualization of adjacent anatomic structures, such as the coronary sinus and coronary arteries, which cannot be easily demonstrated by other imaging modalities. Several studies compared the diagnostic accuracy of CT with that of echocardiography and intraoperative findings. They found sensitivity and specificity of 84%–96% and 93%–100%, respectively, compared with echo and had excellent agreement with operative findings in defining the culprit scallop of the valve [48,49,50,51]. Other studies failed to demonstrate high correlation with operative findings in demonstrating the prolapsing segment [52]. Additional advantages of CT include the ability to demonstrate thicker valve leaflets compared with controls and the ability to map flailing segments. However, there is no study showing that CT can discriminate between myxomatous degeneration and FED. Also, CT cannot help in tissue characterization and, unlike MRI, it cannot demonstrate myocardial pathology. CT has several other disadvantages compared to the other imaging modalities. CT utilizes ionizing radiation. Radiation exposure ranged in one study from 9–11 mSv [48]. Because systolic images are needed for MVP demonstration (in contrast with coronary angriogrphy where radiation exposure can be limited in many cases to diastole only), radiation reduction techniques cannot be used. While CT is suitable to evaluate left ventricular volume very accurately, it cannot be used to estimate the severity of regurgitation. Thus, although MVP imaging with CT is possible, it should not be used as the preferred imaging modality, unless other clinical information, such as coronary evaluation, which can be provided by CT is needed.

## 5. Nuclear Imaging

Patients with MVP often complain of palpitations and chest discomfort. These symptoms have led to a notion of increased prevalence of anxiety disorders in MVP patients. This has been referred to as “mitral valve prolapse syndrome” [53]. The association between MVP and anxiety disorders is currently doubted [54]. Several studies of thallium myocardial perfusion scans on MVP patients demonstrated evidence for ischemia in these patients. These studies suggested a possible explanation for the increased prevalence of chest pain and palpitations. The results were conflicting. While some studies showed evidence for ischemia in patients with normal coronaries, particularly at the insertion point of the pupillary muscle, others showed no evidence for it [54,55,56,57]. These changes were considered to be related to either ischemia of small vessels or fibrosis in the hypo perfused areas [56]. Because of inconsistent findings in these various studies, it is difficult to conclude whether ischemic changes truly exist in MVP patients and what is their clinical significance. Nuclear imaging is routinely used for quantification of chamber size and function, because they are important for clinical evaluation of the patients.

## 6. Conclusions

MVP is diagnosed by imaging modalities by demonstrating valve leaflets ascending into the left atrium passing over the two high points of the saddle shaped annulus into the left atrium during systole. Echocardiography has a central role in diagnosis of MVP and is used as the main tool for this purpose. High special resolution imaging modalities such as MRI and CT can also demonstrate the anatomical relation between the mitral valve annular line and leaflet excursion needed for appropriate diagnosis. The now well-established criteria for definition of MVP based on proper understanding of the mitral valve three-dimensional anatomy and shape has allowed better definition of the disease culminating in finding gene and biological pathways leading to myxomatous degeneration and prolapse. Pathological findings in MVP are not confined to the valve tissue only. Some imaging modalities, such as MRI and, to a lesser, extent nuclear imaging, have demonstrated pathologies in muscle tissue of MVP patients. The pathological correlates of these findings, however, as well as their clinical significance are currently uncertain.
